# Germans and Others behind the Age

**Published:** 1918-04-20

**Authors:** 


### MORE SCOTTISH ASYLUM REPORTS.

### Germans and Others Behind the Age.

Dr. Alexander, of the Kingseat Asylum—which, by the way, he erroneously calls a mental hospital —at Inverness, sends his annual reports for 1913-15-16, and we are glad to find in them some of those educative comments upon insanity whose desirability we have insisted on. It is highly desirable that the medical officers of asylums should utilise the opportunity afforded by their annual reports of educating the public in the many problems that cluster round the subject of insanity; but some of Dr. Alexander's teaching we are unable to endorse. He questions the expediency of finding a cure for general paralysis, and questions it on the ground that in the great majority of cases the disease is contracted by a lapse of self-control, and that by furnishing a cure for the disease the fear of contracting it is lessened. We cannot think that Dr. Alexander really holds an opinion so utterly opposed to the ethics not only of the medical profession, but to those of Christianity itself. What has a doctor to do with the circumstances in which the patient's disease was contracted? Is the doctor to constitute himself a *censor morum*, and to refuse to treat any case if in his opinion the disease was contracted in circumstances of which he disapproves, or in pursuit of an object of which he disapproves? If so, he must refuse to treat not only general paralysis, but also nearly every case of syphilis and of gonorrhœa. If so, he must refuse to treat not only the burglar who falls through a skylight and breaks his leg in the pursuit of his profession, but also the wretched patient who has attempted suicide and failed to carry out his purpose. He must refuse to treat his wounded enemy, and must leave him to die on the field. He must refuse to treat the victim who has been hanging on to the tail of a cart and drops off it, to be run over by a following cart. Nay, before he undertakes the treatment of any case whatever he must inquire into the motives which led the patient into the conduct that caused or contributed to the disease, and decide to treat or not to treat the patient according to the ethical complexion of these motives. He will refuse to relieve the consequences of over-eating, and will leave the drunkard to die of *delirium tremens*. Dr. Alexander has not thought the matter out.

Another bone that we have to pick with him is his adoption of the German term and the German doctrine of *dementia præcox*. It was a Scotchman, Sir Thomas Clouston, who identified this disease many years before Kræpelin "translated" the discovery and brought it forward under another name as his own. Dr. Alexander speaks of the disease, which Clouston called adolescent insanity and Kræpelin called *dementia præcox* as an irrecoverable disease. It is nothing of the kind. Those who have followed our account of the life of Lord Lister know that at the age of about twenty Lister suffered from a sharp attack of insanity, such as is now, when occurring in young people of that age, invariably called by the Germanised school of alienists *dementia præcox*. Such cases are now always so denominated, and a hopeless prognosis is usually given. How ridiculously erroneous this German teaching is is sufficiently proved by Lister's subsequent career. The Germans have posed before the world for many years as the ultra-scientific nation, and they have been accepted, especially by alienists, at their own valuation, in spite of the fact that no scientific discovery of the first rank has ever been made by a German. We hope that the exposure, which has been brought about by this war, of the hollowness of the German pretensions will put an end to this superstition, even among alienists. The Germans had many years' start of us in the preparations for this war, and in every application of science to the art of war the English and French have beaten them. Their Zeppelins are beaten, their aeroplanes are beaten, so that they are constrained to make slavish copies of ours; they began the use of poisonous gas, and we have beaten them at it; their submarine warfare is a failure; they were appalled by our invention of Tanks, and are constrained to imitate them; their artillery is inferior in accuracy and is not as well served. On every point of application of science to warfare we have beaten them, and we had beaten them long before the war in our investigation into insanity. Yet our alienists received them with open-mouthed adulation. It is time the imposture was ended. The term *dementia præcox* was adopted by English alienists as a sign and a proof that they were up to date and in the forefront of the most recent knowledge It is now a proof that they are behind the age.

With Dr. Alexander's other teaching we have no quarrel, and whether we agree or not with what he teaches, we welcome his use of his annual reports as a means of introducing among the lay public discussion of the important problems connected with insanity.

